# Socioeconomic Inequalities in Health-Related Quality of Life among Patients with Cardiovascular Diseases in Vietnam

**DOI:** 10.1155/2018/2643814

**Published:** 2018-09-26

**Authors:** Bach Xuan Tran, Mackenzie PI Moir, Thao Phuong Thi Thai, Long Hoang Nguyen, Giang Hai Ha, Thu Hong Thi Nguyen, Nu Thi Truong, Carl A. Latkin

**Affiliations:** ^1^Institute for Preventive Medicine and Public Health, Hanoi Medical University, Hanoi, Vietnam; ^2^Bloomberg School of Public Health, Johns Hopkins University, Baltimore, MD, USA; ^3^School of Public Health, University of Alberta, Alberta, Canada; ^4^Department of General Planning and Department of Cardiology, Friendship Hospital, Hanoi, Vietnam; ^5^Center of Excellence in Behavioral Medicine, Nguyen Tat Thanh University, Ho Chi Minh City, Vietnam; ^6^Institute for Global Health Innovations, Duy Tan University, Da Nang, Vietnam; ^7^Hanoi Heart Hospital, Hanoi, Vietnam

## Abstract

**Purpose:**

This study aims to explore the sociodemographic differences in health-related quality of life (HRQOL) among Vietnamese patients with cardiovascular diseases (CVD).

**Methods:**

A cross-sectional survey of 600 cardiovascular disease patients (300 inpatients and 300 outpatients) being treated at the Hanoi Heart Hospital was completed between July and December 2016. Data about HRQOL were collected by using the EuroQol-5 Dimensions-5 Levels (EQ-5D-5L) and EuroQOL-visual analogue scale (VAS). Sociodemographic characteristics were collected. A multivariate Tobit regression was used to detect the correlations between HRQOL and sociodemographic factors.

**Results:**

Our sample had an average EQ-5D index of 0.82 (SD=0.21) and VAS score of 77.8 (SD=13.6). Participants were most likely to report problems in pain/discomfort (38.8%) and anxiety/depression (35.2%) and were least likely to report problems related to self-care (19.8%). Age and sex were strongly associated with the EQ-5D index and the VAS. Having health insurance and the number of hospital visits were negatively associated with HRQOL, while participation in the chronic disease management program had the positive relationship.

**Conclusions:**

HRQOL among patients with CVD was moderately lower compared to the Vietnamese general population. Sociodemographic characteristics were strongly associated with HRQOL suggesting that addressing these inequalities should be prioritized in delivering services. Patients should also be encouraged to participate in the chronic disease management program due to its positive effects on quality of life.

## 1. Introduction

Cardiovascular disease (CVD) refers to a group of vascular pathologies including the heart, the brain (cerebrovascular), and blood vessels [1]. CVD is one of the leading causes of mortality worldwide with 17.6 million deaths in 2016 [1, 2]. Up to 90-94% of CVD and stroke cases are attributable to a set of potentially modifiable risk factors like smoking, obesity, psychosocial status, serum lipid levels, hypertension, diabetes, diet, alcohol consumption, and physical activity [3, 4]. Socioeconomic disparities in the risk and treatment of CVD are also well documented [5], generating equity concerns regarding global burden and mortality [6, 7].

CVD is the number one cause of death in Vietnam [8–12]. In 2010, CVD was responsible for 37% of deaths attributable to noncommunicable diseases [11]. Age remains a strong predictor of CVD related deaths in the country and is responsible for the majority of mortalities among the aged and the elderly (60-80+) [9, 10, 13]. Vietnamese men bare the majority of premature deaths and years lived with disability that is attributable to NCD and CVD [11]. Among older adults, CVD is responsible for the majority of disease-related burdens and disabilities. Older females, however, are more likely to suffer from hypertension and heart disease, with longer life expectancy resulting in more years lived with disability when compared to men [13]. Previous work has also highlighted the important role of sociodemographic factors such as age, sex, and geographic location and occupation in the clustering of established CVD related risk factors in Vietnam [14]. A number of social and economic factors have also been demonstrated to play a role in the development of health-related inequality and CVD related morbidity over the life course [15].

While the overall prevalence and burden of CVD in Vietnam are described well in national reports [11, 13], there continues to be a dearth of literature attempting to elucidate its extensive morbidity, particularly as it relates to health-related quality of life (HRQOL). Work outside of Vietnam has examined the HRQOL of patients who have coronary artery disease (CAD) [16], congestive heart failure (CHF) [17], hypertension [18], and stroke [19]. In the absence of this work, HRQOL studies will play an important role as researchers attempt to describe the morbidity and suffering associated with cardiovascular disease in Vietnam. Generating a better understanding of this burden may be crucial when developing effective CVD rehabilitation programs, which have had demonstrable impacts on improving the HRQOL of patients in both Singapore [20] and Malaysia [21]. While previous reports have described the relationship between the development of CVD/ CVD risk factors and sociodemographic factors, what is less clear is how the HRQOL impacts of CVD are related to or determined by these factors. This lack of clarity has resulted in a knowledge gap concerning how the morbidity of CVD manifests within the Vietnamese population based on its sociodemographic characteristics. Evidence of this kind is crucial in the creation of interventions designed to improve the HRQOL of those affected based on these criteria.

The use of generic measures of HRQOL, like the EuroQOL EQ-5D, represents an important metric that is part of a broader effort to build a complete picture of the impact of emerging chronic and cardiovascular diseases in Vietnam. Moreover, health utility measured by the EQ-5D plays a major part in calculating quality-adjusted life years (QALYs), which is a critical component of economic evaluations. As a generic measure, the EQ-5D can capture physical, mental, and social functioning across a range of diseases, treatments, and health interventions [22] and consolidated into a single comparable index [23]. In Vietnam, there has been little work done attempting to measure and compare the HRQOL in patients suffering from cardiovascular disease and its relationship with different sociodemographic characteristics. Ensuring equitable access to treatment and of HRQOL outcomes is an important objective of universal health coverage. This study attempts to measure the sociodemographic differences of HRQOL among patients with CVD in Hanoi, Vietnam.

## 2. Methods

### 2.1. Study Designs and Participants

Our team conducted this cross-sectional study from July to December 2016 at the Hanoi Heart Hospital. We invited patients who had been examined and treated for any cardiovascular diseases to participate in the study, who met following criteria: (1) being able to respond to the questionnaire; (2) being accepted to enroll into the study; and (3) using services in the hospital as inpatients or outpatients. We excluded patients who were unable to respond to the questionnaire. We listed and randomly selected eligible subjects, provided information about the study, and invited them to take part in the survey. Patients who enrolled were asked to give written informed consent. Recruitment efforts drew from 7 departments in the Hanoi Heart Hospital. A total of 600 patients (300 outpatients and 300 inpatients) enrolled in the study.

### 2.2. Measures and Instruments

Data collection team included students at the Hanoi Medical University. Face-to-face interviews were performed using a structured questionnaire. Data about sociodemographic characteristics were collected including age, living area, educational attainment, sex, health insurance status, whether they participated in the chronic diseases management program offered by the hospital, and the number of hospital visits they had in the previous 12 months.

#### 2.2.1. HRQOL

The EuroQol-5 Dimensions-5 Levels (EQ-5D-5L) was utilized to measure the HRQOL of patients. This tool consists of five domains: mobility, self-care, usual activities, pain/discomfort, and anxiety/depression. Each of these domains has five response options: no problems, slight problems, moderate problems, severe problems, and extreme problems/unable to do. A total of 3125 health states are possible via the combination of these domains [24]. By using a cross-walk value set of Thailand, 3125 single indexes corresponding to 3125 health states can be generated, with a score from -0.451 to 1.000 [24]. We also used a visual analogue scale (EQ-VAS) to measure the global rating. This tool has a score ranging from 0 (the worst health you can imagine) to 100 (the best health you can imagine) [25]. A review of the EQ-5D supports its use when investigating the burden of CVD [26], as evidenced by its convergent validity with other generic and disease-specific measures in acute coronary syndrome and stroke [27–29] while showing some discriminative validity in heart failure patients [30]. The version of the EQ-5D used in this study was translated into Vietnamese and previously validated in other HRQOL studies within the country [25].

#### 2.2.2. Statistical Analysis

P value <0.05 was considered statistically significant. We described the socioeconomic status, EQ-5D-5L profiles, utility score, and VAS scores of participants in detail. Significant relationships between items on the sociodemographic questionnaire and the different HRQOL domains (dependent variable) of the EQ-5D were determined using a multivariate Tobit regression.

### 2.3. Ethics Approval

Study protocol was approved by the IRB of the Institute for Preventive Medicine and Public Health, Hanoi Medical University. Patients were invited to participate in the survey during their health care service visits. The purpose of the study, including the benefits and drawbacks of participation, was explained to participants. Participants were also informed that they could withdraw at any time they wanted and that withdrawing from the study would not affect their clinical care in any way. We ensured the confidentiality of participants throughout the study.

## 3. Results

Table 1 contains the reported socioeconomic data from both participant groups. Our cohort had an average age of 57.2 years. There was a pronounced gender disparity within our outpatient group, who were 70.3% female. The majority (65.5%) of our cohort reported that they did not complete high school. While most participants reported having health insurance (82.7%), the majority (64.7%) were not actively participating in the chronic disease management program at the Hanoi Heart Hospital.

We compared responses to the experience of problems within the domains of the EQ-5D (Table 2). Most patients reported having “no problems” in all 5 domains. Participant groups reported significant differences between patient groups were reported within the self-care (p<0.01), pain/discomfort (p=0.04), and anxiety/depression (p=0.02) domains. Participants were most likely to report problems related to pain (38.8%) and were least likely to report problems related to self-care (19.8%). The mean EQ-5D index scores did not differ between inpatient and outpatient groups; however significant differences were found using the EQ-5D VAS (p=0.02).

Relationships between sociodemographic variables and HRQOL are presented in Table 3. We found an association between participant scores on both of our HRQOL instruments with participant age (p<0.01). Similarly, gender had a strong association with index scores from the EQ-5D (p=0.01) and VAS (p<0.01). While some minor internal differences existed within the remaining sociodemographic variables (e.g., education level), these were not related to the EQ-5D or the EQ-5D VAS.

Our analysis found several relationships between HRQOL and sociodemographic variables within our inpatient and outpatient participant groups (Table 4). When compared to those under 30, inpatients between the ages of 30-60+ years reported significantly lower overall HRQOL on the EQ-5D index and on the VAS. Age had a much weaker relationship with HRQOL among our outpatient group. The only significant association (p<0.05) between age and HRQOL among this group was older outpatients (>60 and above) reporting a lower VAS score when compared to their younger counterparts (<30). While males reported having a higher overall HRQOL when compared to females in both groups, this difference was only significant (p<0.05) among outpatients. Surprisingly, no differences were attributable to education level or location. While there was an association between the number of hospitals (p<0.01) and lower HRQOL among inpatients, outpatients participating in the chronic disease management program reported a significantly (p<0.01) higher HRQOL than those who were not.

## 4. Discussion

This study offers needed evidence about the connection between HRQOL and CVD in Vietnam. Our results indicate a moderate impact of CVD on HRQOL among patients and an association between HRQOL and several sociodemographic variables. These results reaffirm the importance of sociodemographic characteristics when studying HRQOL and offer insights into how the burden of CVD manifests within the Vietnamese population. A measure of this burden, alongside their relationship with specific sociodemographic variables, is critical to efforts aimed at improving outcomes of CVD related care in Vietnam.

We found an average EQ-5D index of 0.82 and VAS of 77.8, which were lower than those of the general Vietnamese population [31], including elderly and rural populations [32, 33]. Our index was similar to those living in the country's mountainous regions [34]. We observed a higher EQ-5D index compared to previous work investigating HRQOL impacts of CVDs associated with severe symptoms and functional limitations such as chronic stable angina (0.75 and 0.712) [35]. While Azmi et al. (2015) reported a lower index in acute coronary syndrome patients at baseline (0.75 and 0.60), these patients reported an index of 0.82 and VAS of 0.71 after 12 months of participation in a cardiac rehabilitation program [36], similar to that reported by our cohort. For a local comparison, our EQ-5D index and EQ-VAS were higher than Tran et al.'s 2015 study in both Vietnamese male (0.68 and 0.657) and female (0.67 and 0.623) patients at three months after stroke. When compared to our results, other work has reported a lower index and VAS score among patients who have suffered from a heart attack [29] and stroke [28, 37]. While we reported a higher HRQOL index and VAS when compared to patients from several countries who had multiple cardiovascular events (0.70 and 61.0), our index results were similar to those who had suffered from an initial event but did not experience subsequent events (0.80 and 68.2) [38].

Our cohort reported an array of problems within the 5 domains of the EQ-5D. Similar to previous work, our patients were least likely to report problems in the self-care domain [19, 35, 39] and were most likely to report having problems related to pain [19, 39]. Hospitalized patients were more likely to report problems in the self-care domain when compared to our outpatient group. This is likely due to a higher prevalence of physical impairment among those who clinically require their care be provided within the hospital and need assistance with activities of daily living. Meanwhile, outpatients in our cohort were more likely to report problems within the pain and anxiety/depression domains. One explanation for these phenomena is that, alongside pain/discomfort, patients may also be worried about their disease severity and its related social and economic consequences. Higher reporting of problems related to pain is a common finding among the aged and the elderly in Vietnam [32, 33]. The gender disparity present among outpatients (70.3% female) may also explain why this group was significantly more likely to report issues related to mental health, anxiety/depression, and pain [33, 34, 40–42].

In this study, we found associations between age, gender, and HRQOL. Specifically, there was a significant relationship between older age and lower HRQOL, especially among hospitalized patients. The impact of age on HRQOL in CVD populations using the EQ-5D is mixed. While some studies examining CVDs have found an association between the EQ-5D index and increasing age [39, 43], others have not [35, 36]. Meanwhile, regarding gender, males reported a higher EQ-5D utility index and VAS score than their female counterparts. As was the case with age, the evidence for sex-based disparities in HRQOL among CVD patients is mixed using the EQ-5D. Some work has found higher utility scores among males at baseline [36] whereas other work found that HRQOL disparities between both sex and age groups were durable despite the impact of stroke [37]. In contrast, other work examining a range of CVDs did not find an association between sex and health-related quality of life using the EQ-5D [35, 39, 43]. Women living in the mountainous regions of Vietnam have, however, reported lower overall HRQOL than men [34]. In Vietnam, modifiable CVD risk factors (metabolic and behavioral) have been demonstrated to increase with age, cluster differently by sex, and be related to several sociodemographic variables [14]. In Sweden, the contribution of health-related inequalities to CVD morbidity increases with age, with the contribution of individual socioeconomic variables (e.g., age, income, education, and area of residence) to this inequality shifting over the life course [15]. Together, these findings suggest several confounding variables are responsible for obfuscating the relationship between age, CVD risk and morbidity, and the likelihood of having a lower HRQOL.

We found that education and living area were not related to HRQOL. Previous work studying CVD patients has found an association between education and HRQOL [18, 39, 44]. In contrast, other studies using the EQ-5D have not found an association between education and HRQOL, but did find a relationship using a different generic measure [35]. A lower HRQOL has been associated with living in urban environments and having a higher education within the general Vietnamese population [31], alongside having less education [33, 41]. These results suggest that patients are accessing services and have equal treatment outcomes irrespective of their education and location of residence.

Notably, patients who participated in the outpatient chronic disease management program reported higher HRQOL when compared to those who did not. This relationship is similar to previous work documenting HRQOL improvements over time among CVD patients undergoing rehabilitation [36, 45]. The greater psychological burden found in our outpatient group may represent poor emotional coping and distress in response to having to manage their illness and be related to the low attendance of the outpatient chronic disease management program offered by the hospital. Similar challenges with rehabilitation program attendance have been described elsewhere and point out the importance of considering mental health and psychological distress as an explanation for early dropout, given their effectiveness in improving overall well-being among patients [20, 21].

This study has several implications for future research and clinical practice in Vietnam. First, age and gender differences in HRQOL warrant that additional attention is paid to older and female patients when designing health care policy and services. Second, our findings indicate that patients should be encouraged to participate in outpatient chronic disease management programs, which may sustainably improve and maintain their HRQOL. Third, our findings suggest that further work be done to examine the effectiveness of current outpatient management programs and how to increase their enrollment.

There were several limitations to this study. First, the cross-sectional nature of the design did not allow us to capture HRQOL improvements over time, thereby limiting its generalizability. Second, our lack of disease-specific data was a major limitation and prevented us from capturing the varied functional and HRQOL impacts of different cardiovascular diseases. Collecting general data on CVD therefore limited the interpretation of these results, including its comparability with other disease-specific HRQOL studies. A third limitation was our lack of captured data regarding other important covariates that may determine HRQOL within this population. For example, we did not ask participants about income, affordability of medication, travel distance to the hospital (for outpatients), marital status, or if they had an informal caregiver helping them manage their care.

## 5. Conclusion

We found that HRQOL among patients with CVD was lower when compared to the general Vietnamese population. Age and sex were strongly associated with HRQOL and should be considered as important to the effective delivery of care and other health care services. The higher HRQOL among those who enrolled in the outpatient chronic disease management program suggests that patients should be encouraged to participate in similar programs in order to maintain their well-being and maximize other related outcomes.

## Figures and Tables

**Table 1:** Demographic characteristics of respondents (n=600).

	**n**	%
**Gender**		
(i) Male	249	41.5
(ii) Female	351	58.5
**Education**		
(i) < High school	393	65.5
(ii) High school	110	18.3
(iii) > High school	97	16.2
**Living location**		
(i) Urban	280	46.7
(ii) Rural	320	53.3
**Having health insurance**		
(i) Yes	496	82.7
(ii) No	104	17.3
**Participating in the chronic diseases management program in hospital **		
(i) Yes	212	35.3
(ii) No	388	64.7
**Types of patients**		
(i) Outpatient	300	50.0
(ii) Inpatient	300	50.0

	**Mean**	**SD**

**Age**	57.2	19.9
**Number of hospital visits last year**	4.4	4.7

**Table 2:** Health-related quality of life in patients with cardiovascular diseases.

**Domain**	**Outpatients**		**Inpatients**		**Total**		**p value**
	**N**	%	**N**	%	**N**	%	
**Mobility**							
No problems	231	77.0	220	73.3	451	75.2	0.30
Have problems	69	23.0	80	26.7	149	24.8	
**Self-care**							
No problems	254	84.7	227	75.7	481	80.2	<0.01
Have problems	46	15.3	73	24.3	119	19.8	
**Usual activities**							
No problems	242	80.7	222	74.0	464	77.3	0.51
Have problems	58	19.3	78	26.0	136	22.7	
**Pain/Discomfort**							
No problems	171	57.0	196	65.3	367	61.2	0.04
Have problems	129	43.0	104	34.7	233	38.8	
**Anxiety/Depression**							
No problems	176	58.7	213	71.0	389	64.8	0.02
Have problems	124	41.3	87	29.0	211	35.2	

	**Mean**	**SD**	**Mean**	**SD**	**Mean**	**SD**	**p value**

**EQ-5D index**	0.82	0.20	0.81	0.22	0.82	0.21	0.76
**EQ-VAS**	76.5	12.7	79.1	14.3	77.8	13.6	0.02

**Table 3:** EQ-5D index and EQ-VAS according to socioeconomic characteristics of patients.

**Domain**	**Total**		**EQ-5D index**			**EQ-VAS**		
	**n**	%	**Mean**	**SD**	**p-value**	**Mean**	**SD**	**p-value**
**Age groups**								
< 30 years old	56	9.3	0.93	0.16	<0.01	90.1	10.1	<0.01
30- <45 years old	55	9.2	0.85	0.17		78.3	13.9	
45- <60 years old	167	27.8	0.85	0.20		78.3	12.4	
≥ 60 years old	322	53.7	0.77	0.22		75.3	13.5	
**Gender**								
Male	249	41.5	0.84	0.20	0.01	79.7	13.5	<0.01
Female	351	58.5	0.80	0.22		76.5	13.5	
**Education**								
< High school	393	65.5	0.81	0.21	0.91	78.0	13.6	0.88
High school	110	18.3	0.82	0.21		77.6	13.2	
> High school	97	16.2	0.82	0.21		77.3	14.2	
**Living location**								
Urban	280	46.7	0.82	0.21	0.59	77.1	13.7	0.26
Rural	320	53.3	0.81	0.21		78.4	13.4	
**Having health insurance**								
Yes	496	82.7	0.81	0.21	0.16	77.9	13.9	0.65
No	104	17.3	0.84	0.19		77.2	11.9	
**Participating in the chronic diseases management program in hospital **								
Yes	212	35.3	0.82	0.21	0.71	76.9	13.8	0.22
No	388	64.7	0.81	0.22		78.3	13.4	

**Table 4:** Factors associated with EQ-5D index and EQ-VAS in patients with cardiovascular diseases.

Characteristics	EQ-5D index				EQ-VAS			
	Outpatients		Inpatients		Outpatients		Inpatients	
	Coef.	95%CI	Coef.	95%CI	Coef.	95%CI	Coef.	95%CI
**Age groups (vs < 30 years old)**								
30 - < 45 years	-0.004	-0.33; 0.33	**-0.50** ^∗∗∗^	-0.75; -0.26	-8.21	-19.98; 3.56	**-16.66** ^∗∗∗^	-24.43; -8.88
45 - < 60 years	-0.02	-0.33; 0.30	**-0.40** ^∗∗∗^	-0.60; -0.20	-8.25	-19.43; 2.94	**-16.69** ^∗∗∗^	-22.70; -10.67
60 years and above	-0.13	-0.44; 0.18	**-0.55** ^∗∗∗^	-0.74; -0.36	**-12.12** ^∗∗^	-23.18; -1.05	**-18.72** ^∗∗∗^	-23.84; -13.60
**Gender (Male vs Female)**	**0.12** ^∗∗^	0.02; 0.22	0.09^∗^	-0.01; 0.19	**3.66** ^∗∗^	0.28; 7.03	2.36	-1.18; 5.90
**Education (vs < High school)**								
High school	-0.04	-0.14; 0.07	0.03	-0.12; 0.18	-1.33	-5.05; 2.40	2.95	-2.51; 8.40
> High school	-0.01	-0.14; 0.13	-0.01	-0.15; 0.13	-1.02	-5.83; 3.80	0.94	-3.96; 5.84
**Living location (Urban vs Rural)**	0.07	-0.02; 0.16	0.04	-0.06; 0.15	-1.70	-4.86; 1.46	-0.12	-3.89; 3.66
**Having health insurance (Yes vs No)**	**-0.19** ^∗∗∗^	-0.32; -0.06	-0.02	-0.23; 0.20	-1.36	-6.01; 3.28	-7.36^∗^	-15.26; 0.55
**Number of hospital visits last year**	0.002	-0.01; 0.02	**-0.08** ^∗∗∗^	-0.14; -0.03	0.21	-0.29; 0.71	-0.99	-2.97; 0.99
**Participating in the chronic diseases management program in hospital (Yes vs No)**	**0.19** ^∗∗∗^	0.05; 0.34	-0.03	-0.16; 0.10	2.23	-2.85; 7.32	-2.48	-7.37; 2.41
**Constant**	0.99^∗∗∗^	0.68; 1.29	1.47^∗∗∗^	1.17; 1.78	85.35^∗∗∗^	74.36; 96.34	102.21^∗∗∗^	92.39; 112.03

^*∗∗∗*^ *p<0.01,* ^*∗∗*^ *p<0.05,* ^*∗*^ *p<0.1*.

## Data Availability

The data that support the findings of this study are available from the Institute for Preventive Medicine and Public Health, Hanoi Medical University, Hanoi, Vietnam, but restrictions apply to the availability of these data, which were used under license for the current study, and so are not publicly available. Data are however available from the authors upon reasonable request and with permission of the Institute for Preventive Medicine and Public Health, Hanoi Medical University, Hanoi, Vietnam (Prof. Bach Xuan Tran, bach.ipmph@gmail.com).
